# Biomonitoring of Silver in Children and Adolescents from Alcalá de Henares (Spain): Assessing Potential Health Risks from Topsoil Contamination

**DOI:** 10.3390/toxics13121026

**Published:** 2025-11-27

**Authors:** Antonio Peña-Fernández, Manuel Higueras, Roberto Valiente, M. Carmen Lobo-Bedmar

**Affiliations:** 1 Department of Surgery, Medical and Social Sciences, Faculty of Medicine and Health Sciences, University of Alcalá, Ctra. Madrid-Barcelona, Km. 33.600, Alcalá de Henares, 28871 Madrid, Spain; 2 Scientific Computation Research Institute (SCRIUR), Universidad de La Rioja, La Rioja, 26006 Logroño, Spain; 3 Department of Geology, Geography and Environment, University of Alcalá, Calle Colegios 2, Alcalá de Henares, 28801 Madrid, Spain; 4 Madrid Institute for Rural, Agricultural and Food Research and Development (IMIDRA), Department of Agri-Environmental Research, Finca el Encín, Crta. Madrid-Barcelona, Km. 38.2, Alcalá de Henares, 28800 Madrid, Spain; carmen.lobo@madrid.org

**Keywords:** silver, hair biomonitoring, children, adolescents, reference values, ICP-MS, urban exposure, Spain, topsoil contamination

## Abstract

This study assessed exposure to silver (Ag) in children and adolescents living in Alcalá de Henares, an urban-industrial city in the Madrid Region (Spain), in light of the growing use of Ag in consumer and medical products. Scalp hair was collected from 120 children (6–9 years; 70 females) and 97 adolescents (13–16 years; 68 females) permanently residing in the city, and Ag concentrations were determined by inductively coupled plasma mass spectrometry under strict quality control. In parallel, 97 topsoil samples from urban parks were analysed to evaluate potential environmental sources, and risks of non-carcinogenic exposure via ingestion and dermal routes were characterised. Median Ag levels (µg/g) were 0.1205 in girls and 0.0892 in boys among children, and 0.1057 and 0.0387 among adolescent females and males, respectively. No significant differences were observed between age groups (*p* = 0.153), but females showed consistently higher levels than males. Proposed reference intervals (CI-PP95) were 0.2866–0.5383 µg/g for children and 0.5248–3.0795 µg/g for adolescents. Hazard quotients for soil exposure were below unity, indicating minimal health risk. Overall, Ag exposure in Alcalá’s youth was low and consistent with background levels in non-occupationally exposed populations. The higher values found in some female adolescents likely reflect lifestyle-related sources. These results provide the first local reference values for Ag in hair of Spanish children and adolescents, offering a baseline for future biomonitoring and temporal trend analyses.

## 1. Introduction

Silver (Ag) is used extensively in a wide variety of personal care products and industrial products. Current applications include the synthesis of silver nanoparticles and engineered nanomaterials in an increasing number of products including water filters, paints, cosmetics and detergents, textiles, food packaging and plastics, medical products and devices, electrical appliances, and biosensors [1,2,3]. Despite the relatively low toxicity of Ag to humans, as suggested by its low position within the Agency for Toxic Substances and Disease Registry (ATSDR) 2022 Substance Priority List (number 227 [4]), the increasing use of Ag in pharmaceutical and personal care products, as well as in electronic and nanomaterial products, may result in greater human exposure and associated health risks [5]. Elevated exposure to Ag has been linked to neurological and psychiatric clinical outcomes [6] and can disrupt the metabolic processes of essential elements, such as selenium [5]. Consequently, the estimation of exposure to Ag in non-occupationally exposed populations has become increasingly relevant to address potential adverse effects arising from its expanding exposure sources [1].

Recent years have seen increased interest from the European Union (EU) in the development of large Human Biomonitoring (HBM) studies across Europe. Hair has been identified as a useful, non-invasive biomarker for HBM, since metal cations bind to sulphur molecules within keratin [7]. This is particularly relevant when assessing younger and environmentally sensitive age groups. However, there remain limitations, including a paucity of reference ranges and inconsistent correlations with other biomarkers [7].

In Spain, there have been few HBM studies of metals and metalloids in hair on normal/healthy populations, and those that are available have been limited to adult cohorts and/or potentially contaminated or industrial areas. Furthermore, the majority of HBM studies conducted on the Spanish youth population have been restricted to the analysis of a few of the so-called heavy metals, such as lead, cadmium, and mercury. This limitation is even more pronounced in relation to young individuals residing in the Madrid Region, Spain. To the best of our knowledge, there is only one study that has reported levels of silver in scalp hair from children aged 0–18 years residing in Madrid city [8]. Given that Madrid and its metropolitan area constitute one of Spain’s largest and most industrially and demographically developed regions, assessing Ag exposure in this setting provides a valuable reference for understanding background levels in densely urbanised European environments [7,8]. However, internationally available data on Ag in human hair are limited and highly variable, with studies from Asia, Eastern Europe, and the Americas generally reporting higher concentrations than those observed in Spain [5,6,7,8].

Biomonitoring for Ag has been identified as a method to overcome the challenges associated with estimating exposure to this metal. The potential for exposure to Ag is multifaceted, with multiple and varied routes, including oral (e.g., through food), inhalation and dermal (e.g., through textiles or personal care products that contain Ag nanoparticles due to its antibacterial properties). Additionally, Ag exists in diverse chemical forms (including elemental, ionic and nanoparticle forms) [1,2]. In light of the challenges associated with the estimation of exposure to Ag, there is an ongoing effort to identify suitable human matrices and detection and quantification methods. This endeavour is aimed at facilitating a comprehensive study of human exposure to Ag, thereby addressing potential risks to humans posed by the rapid increase in the use of various Ag forms, including nanoparticles [2]. Human hair has been identified as a suitable non-invasive matrix for biomonitoring Ag, as this metal can interfere with biological components present in the urine [5]. In this study, hair Ag is therefore interpreted solely as an indicator of external environmental and lifestyle-related exposure, rather than as a biomarker of internal dose. This is particularly relevant given that the HBM4EU initiative has classified Ag nanoparticles as a Category D substance—indicating toxicological concern but limited HBM data—and highlighting the need to generate first-level exposure information for Ag [9].

Notwithstanding the growing public health concern that Ag is attracting, particularly with regard to Ag nanoparticles, only a select number of comprehensive national-level HBM programmes incorporate Ag as a chemical to biomonitor in their studies, namely the Canadian Health Measures Survey (CHMS, [10]) and the Brazilian Longitudinal Study of Adult Health (ELSA-Brasil [11]). Significantly, major national-level HBM initiatives do not monitor this metal, including the National Health and Nutrition Examination Survey (NHANES) in the United States [12], the German Environmental Surveys (GerESs) [13], the Chinese National Human Biomonitoring (CNHB) [14] and the European HBM initiative (HBM4EU) [15]. It is also important to note that silver has been only rarely monitored in human biomonitoring studies, which means that the limited literature available on Ag concentrations in hair has been published relatively recently and primarily reflects the emerging nature of this research field.

In this context, the present study expands previous research in Alcalá de Henares by providing biomonitoring data for two sensitive groups: children aged 6–9 years and adolescents aged 13–16 years. The inclusion of adolescents allows, for the first time in Spain, a direct comparison of Ag accumulation between younger children and teenagers within the same urban environment. In addition, this work uniquely integrates concurrent analysis of topsoil samples collected from the same city to provide environmental context and explore potential external contributions to exposure. Together, this dual approach advances current knowledge by linking internal (hair) and external (surface soil) exposure indicators within a single study area, thereby strengthening the human biomonitoring evidence base for less-studied elements such as Ag. Therefore, the main objectives of this study were to: (a) characterise Ag exposure in both children and adolescents living in Alcalá de Henares using scalp hair as a biomarker; (b) propose updated reference values for these age groups; and (c) explore potential age- and sex-related differences in Ag deposition, as well as possible links with environmental Ag contamination in topsoils across the city.

## 2. Materials and Methods

### 2.1. Site Characteristics

Alcalá de Henares, also referred to as Alcalá, a World Heritage City located in the central region of Spain, is situated 31 kilometres from Madrid city (accessible via the A-2 motorway), 15 kilometres from Madrid–Barajas international airport. The population of Alcalá was 200,702 as of 2024 (INE, 2025 [16]), making it one of the most populous urban areas in the Comunidad de Madrid, after the capital itself. Alcalá’s demographic prominence in the Madrid Region largely stems from its position as the main economic hub in the Henares Corridor, a historically industrial area that developed along the Henares River, northeast of Madrid.

### 2.2. Scalp-Hair Sampling

Ag was analysed in scalp hair from healthy children (6–9 years old; *n* = 120, 70 females) and adolescents (13–16 years old; *n* = 97, 68 females) residing within Alcalá de Henares municipality. The selected individuals met strict inclusion criteria developed to mitigate the effects of different factors that can affect the elemental content in hair [7]. All participants were of Caucasian ethnicity, had dark untreated hair, no history of hair dye use, and were not receiving medical treatment. Hair strands were cut from the occipital region of the scalp as close as possible to the skin, and approximately the first 1 cm of proximal hair was used for analysis to reflect recent exposure and reduce the influence of hair length.

**Figure 1:**
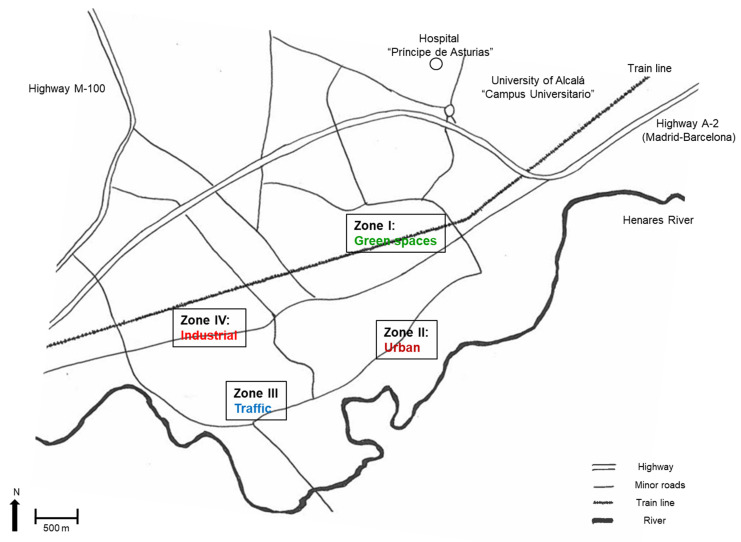
Study area and sampling sites.

We developed a geographically based recruitment strategy to ensure that our sample was stratified across different neighbourhoods of the city, representing varying predominant land uses that might influence Ag exposure. We then divided the city into four Zones (Figure 1). Zone I included low-population-density neighbourhoods characterised by abundant green areas and open spaces and a predominantly residential land use. Zone II encompassed high-population-density areas, with an urban landscape dominated by multi-family housing blocks and a more heterogenous land use (residential and commercial used are mixed). In contrast, Zone III covered all areas of the city with a higher density of car traffic (proximal to major avenues and main roads), and Zone IV included areas with predominantly industrial land uses. We classified the municipal schools into one of these four zones based on their location. Children were recruited from primary schools located in Zones I, II, and IV, while adolescents were recruited from secondary schools in all Zones.

Hair samples were collected between April and May of 2001, providing one of the earliest biomonitoring datasets for silver in Spain. This historical baseline is particularly valuable for future comparisons with more recent cohorts to assess temporal trends in exposure. In addition, the increasing incorporation of silver nanoparticles into textiles, cosmetics, and household products underscores the relevance of analysing these early samples, which establish pre-nanotechnology reference values for children and adolescents in Spain. As such, these baseline values play a critical role in enabling future assessments of whether Ag exposure has evolved over time and in supporting potential public health responses.

The study was conducted in accordance with the Declaration of Helsinki, and written informed consent was obtained from parents or legal guardians of all participants. In the case of adolescents, additional written assent was also obtained from the participants themselves. Hair samples were appropriately collected from the occipital region of the head using stainless steel scissors during face-to-face meetings at the participating schools.

Each hair sample (approximately 1.0 g) was washed with Triton X-100 (1%, *v*/*v*; purchased from Sigma-Aldrich Co., St. Louis, MO, USA) in ultrapure water (Milli-Q^®^ Direct 8, Merck Millipore, Darmstadt, Germany; resistivity 18.2 MΩcm) for 5 min in an ultrasonic bath (J.P. Selecta Ultrasons, Abrera, Barcelona, Spain), and a second time only with ultrapure water Milli-Q, in order to reduce the influence of potential exogenous contamination [17,18]. Following this step, the samples were rinsed with ultrapure water Milli-Q, and dried at approximately 50 °C to a constant weight. Triton X-100 was selected for use due to its established role in the field [17]. Non-ionic surfactants, such as Triton X-100, have been shown to have minimal impact on the hair’s endogenous metal composition, making them a suitable choice when the hair is intended for diagnostic purposes or biomarker-based exposure assessment [17].

Approximately 100 mg of each hair sample was treated with 2 mL of nitric acid 65% (Suprapur; Merck, Darmstadt, Germany) for 8 h at room temperature. Subsequently, the samples were heated at 96 °C for 12 h. The solutions were diluted to 10 mL with ultrapure water Milli-Q and frozen at −80 °C until analysis [19,20]. The concentration of Ag was determined by inductively coupled plasma-mass spectrometry (ICP-MS; PerkinElmer NexION 350D, PerkinElmer Inc., Waltham, MA, USA) operating in collision mode with helium gas (He) to minimise polyatomic interferences, according to previous methods conducted by the authors [7]. ICP-MS has been described in the literature as an appropriate technique for monitoring Ag and it is used to biomonitor Ag in the Canadian Health Measures Survey (CHMS [21]). In summary, each sample was analysed in duplicate, with NCS DC 73347 employed as the quality control standard at an interval of 5 samples [Human hair Ag = 0.029 ± 0.008 µg/g; National Research Centre for Certified Reference Materials (CRMs), No. 7 District 11, Hiepjinge 100013, Beijing, China]. Additionally, blanks—which had been included during the process of acid mineralisation—were also analysed in each set of five samples. The mean recovery rate was approximately 90%. The limits of detection (LoD) were 0.0036 and 0.0224 µg/g, for children and adolescents, respectively (Table 1). The difference in LoD values reflects variations in sample mass and dilution factor rather than analytical quality. Both groups were analysed under identical ICP-MS conditions; however, smaller sample weights obtained after cleaning and trimming of adolescent hair required greater dilution prior to analysis, resulting in a slightly higher instrumental LoD.

This protocol was reviewed and approved by the “Comité de Ética de la Investigación y de Experimentación Animal” (CEI-EA) of the University of Alcalá, which is formally authorised to evaluate both human and animal research (CEI-EA: CEIP/2025/3/089).

### 2.3. Reference Values 

The utilisation of reference levels of metals in human hair remains a contentious subject, owing to the various factors that may influence their presence. Nonetheless, these levels have been proposed in comprehensive surveys conducted in specific countries and/or regions. The levels of Ag found in the group of children and adolescents monitored were compared with the reference levels proposed by Ballesteros et al. [8] for young individuals living in the Madrid region (specifically three educational centres were monitored: two in Madrid city and one in a village in the near northeast): 0.032–1.430 µg/g (0–18 years old).

Potential reference values for Ag in the hair of healthy children and adolescents residing in Alcalá de Henares were also proposed for female and male participants following the recommendations highlighted by the International Union of Pure and Applied Chemistry (IUPAC) [22]. The reference values were calculated using a non-parametric approach, corresponding to the 95th percentile of the population distribution for Ag in the scalp hair (biomarker), with a 95% confidence interval (CI-PP95).

### 2.4. Topsoil Samples

HBM has been demonstrated to be a valuable tool in the assessment of health risks posed by chemicals in the environment. By measuring the actual concentrations of chemicals in the human body (internal exposure), as opposed to external exposure, HBM provides a more comprehensive evaluation of the potential health implications [23]. The human risks associated with the presence and distribution of Ag in topsoils from urban parks across Alcalá de Henares have been characterised in Peña-Fernández et al. [24] *publication in preparation*, and are briefly described here to better contextualise the risks identified for children and adolescents (the most environmentally sensitive groups of the population).

Briefly, a total of 97 topsoil samples (0–3 cm depth) were randomly collected in July 2001 from various urban parks and recreational areas across Alcalá [24,25]. Approximately 0.7 g of topsoil samples that had been pre-processed in accordance with previous methods were treated with 5 mL of nitric acid (65% Suprapur, E. Merck, Darmstadt, Germany) in Teflon bombs for 8 h at room temperature. Each topsoil sample was then heated at 96 °C for 12 h. Following this, the solutions were filtered and made up to 25 mL with ultrapure water Milli-Q. Levels of Ag were also monitored by ICP-MS, following a similar strategy as described for the analysis of this metal in hair. The mean recovery rate was close to 90%, and the limit of detection for this metal was 0.049 µg/g.

The non-carcinogenic human risks associated with exposure to Ag through ingestion and dermal absorption of topsoils were assessed in accordance with the US EPA Risk Assessment Guidance for Superfund (RAGs) methodology [26,27]. Inhalation risks were not quantified, as the US EPA has not yet provided an inhalation reference concentration (RfC) for silver present in resuspended soils yet [28].

Although soil samples were collected from urban parks and recreational areas across Alcalá de Henares, they were not always located near the schools where participants were recruited. Therefore, these data reflect the general urban environmental background rather than specific school or household surroundings. The potential spatial mismatch between soil sampling sites and individual exposure areas is acknowledged and further discussed.

### 2.5. Statistical Analysis

Statistical methods appropriate for censored data (non-detects, i.e., values below the limit of detection, LoD) were applied, following the recommendations of Helsel [29] and Shoari and Dubé [30]. Simple substitution approaches (e.g., replacing non-detects with one-half the detection limit) were avoided, as these may allow imprecise values to disproportionately influence total estimates and compromise risk characterisation. Instead, the following procedure was employed: (a) for <50% censoring, the Kaplan–Meier method was used; (b) for 50–80% censoring, robust regression on order statistics (ROS) was applied for *n* < 50 samples and maximum likelihood estimation (MLE) for *n* > 50; and (c) for >80% censoring, only high sample percentiles were reported. These approaches were implemented using the ‘NADA’ and ‘NADA2’ packages in the R statistical environment, version 4.4.3 [31].

To evaluate inter-area differences in Ag concentrations in hair, the Peto–Peto one-factor test was applied for censored data. For uncensored variables, the choice of statistical test depended on the outcome of normality and homoscedasticity tests. When both assumptions were met (normality assessed via the Shapiro–Wilk test and equal variances via the Fligner–Killeen test), Duncan’s new multiple range test was used. If normality was not rejected but homoscedasticity was rejected, pairwise Welch’s *t*-tests were conducted. If normality was rejected, pairwise Wilcoxon rank sum tests were used instead. Multiple comparison *p*-values were adjusted using the Benjamini–Hochberg false discovery rate procedure.

Correlation analyses were conducted to evaluate the relationships between Ag levels in hair and Ag concentrations in topsoils. Given the skewed distribution of environmental data, non-parametric Spearman’s rank correlation coefficients (ρ) were calculated, with significance levels set at α = 0.05.

## 3. Results

### 3.1. Ag in Hair of Children and Adolescents

As summarised in Table 1, Ag was detected in all children’s hair samples and in most adolescents’ samples (80.4%), with 19.6% of adolescent values being below the limit of detection. Median concentrations in children were 0.1120 (range 0.0139–2.6906), with a geometric mean of 0.1016. Adolescents showed greater dispersion driven by several high values among females (range 0.0225–9.9268; arithmetic mean 0.5401 ± 1.4498), although central tendency (median 0.0695; geometric mean 0.0839) was similar to or slightly lower than that of children (median 0.1120; geometric mean 0.1016); with all values presented as µg/g.

Overall, comparison between age groups indicated higher inter-individual variability among adolescents, with outlier values almost one order of magnitude above those detected in children. Direct comparison of Ag concentrations between children and adolescents did not reveal statistically significant differences (*p*-value = 0.153).

### 3.2. Sex Differences

In children, Ag levels were significantly higher in females compared to males (*p*-value = 0.015). Female children showed a median of 0.1205 (range 0.0168–2.6906), while male children showed 0.0892 (range 0.0139–0.3841). Among adolescents, a similar sex-dependent pattern was observed (*p*-value = 0.002), with female adolescents presenting higher levels (median 0.1057, range 0.0225–9.9268) compared with males (median 0.0387, range 0.0273–0.5023); with all values presented as µg/g.

### 3.3. Residential Areas

Table 2 shows Ag concentrations in hair according to residential area. In children, no statistically significant differences were observed between Zones (*p*-value = 0.172), although slightly higher values were detected in Zone II (high-population-density) compared with Zones I (low-population-density) and IV (industrial).

In adolescents, statistically significant differences were found between Zones (*p*-value = 0.0004). Individuals from Zone I (low-population-density) exhibited the lowest levels (median 0.0320 µg/g), which were significantly different from those in Zones II (high-population-density) and III (traffic-dense), which presented higher median concentration values (0.2145 and 0.0887 µg/g, respectively). Although samples obtained in zone I presented a slightly lower median concentration than ones collected in industrial areas (Zone IV, 0.0695 µg/g), no significant differences were found between these two zones.

### 3.4. Correlations Between Hair and Topsoils

**Figure 2:**
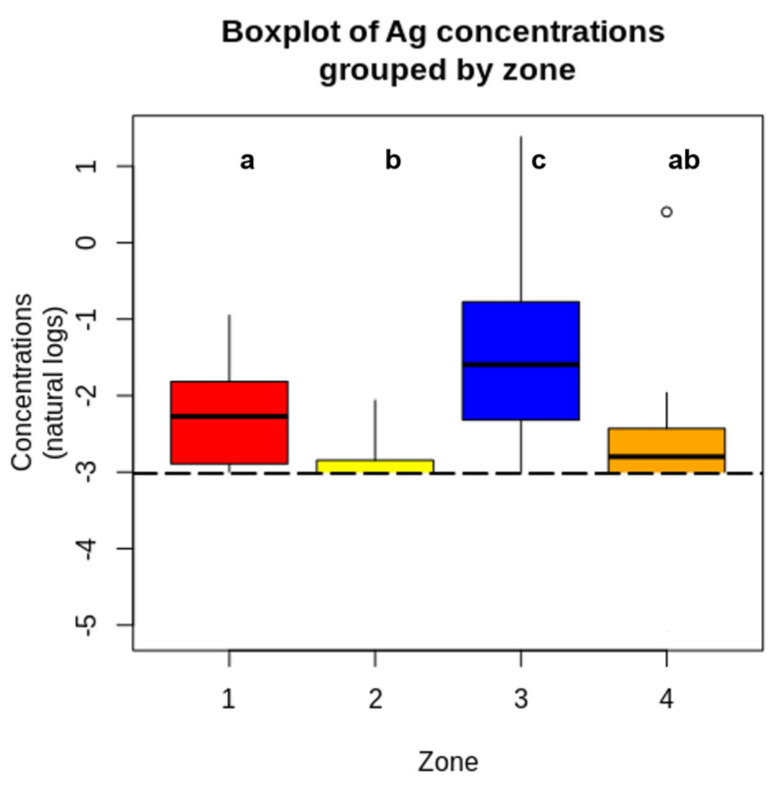
Levels of Ag in topsoils monitored in each main area in Alcalá de Henares. Box-and-whisker plots for Ag concentrations (mg/kg). The line inside the box represents the median value; the boxes mark the 25th and 75th percentiles; the horizontal lines outside the box (whiskers) mark the values that extend 1.5 times the width of the box. Points outside the whisker are called outliers, and these are marked with an extreme symbol if they are more than three times the width of the box. The concentration values (arithmetic mean (µg/g) ± SD) with different letters were significantly different (*p*-value = 0.0000000278). Colours represent statistically distinct groups. Mixed colours (e.g., orange) correspond to groups that share letters (e.g., “ab”), indicating they are not statistically different from either group “a” or “b”.

Figure 2 illustrates the distribution of Ag concentrations in topsoils across the four main areas of Alcalá, highlighting slightly higher levels in compact urban and garden areas compared with industrial and low-density residential zones [24].

To explore possible environmental origins of exposure, Ag concentrations in hair were correlated with Ag concentrations measured in matched surface soil samples collected from urban parks and recreational areas across Alcalá de Henares. At the global level, the Spearman correlation coefficient among children hair samples and soils was very weak and not statistically significant (*r* = −0.06, *p*-value > 0.05). When stratified by sex, correlations also remained non-significant, with *r* = 0.02 in males and *r* = −0.13 in females. Among adolescents, similar findings were observed, with non-significant weak correlations for the general group (*r* = −0.04), male (*r* = 0.02) and female (*r* = −0.06).

Overall, these results indicate that Ag levels in soils are not a primary determinant of Ag accumulation in hair among the populations studied. Instead, alternative exposure pathways, such as airborne particles, indoor dust, dietary intake, or the use of personal and household products containing Ag (including Ag nanoparticles), may play a more important role in explaining the observed inter-individual variability, particularly among adolescents. Localised patterns suggested slightly stronger associations in Zone II, an urbanised area, which may indicate that soils could contribute partially to Ag body burdens in certain environments, although lifestyle and consumer-related exposures are likely more influential. These findings are consistent with the companion study on urban and industrial soils from Alcalá de Henares, where Ag showed clear anthropogenic enrichment across all land-use types, particularly in garden and urban soils, confirming its human-related origin and spatial variability in the city [24].

### 3.5. Risk Characterisation

Hazard quotients (HQs) for Ag, calculated using the soil exposure dataset and methodology described in a companion manuscript currently in preparation [24], were below unity for both children and adolescents, indicating minimal non-carcinogenic risk from topsoil ingestion and dermal contact. In that work, HQs were derived from estimated daily intakes of Ag for each exposure pathway and age group, applying standard exposure parameters and reference values to ensure comparability with other metals analysed in the same soils. This consistent approach provides robust support for the conclusion that Ag exposure through soil contact is negligible in Alcalá de Henares. However, the higher Ag concentrations observed in adolescents’ hair suggest that additional exposure routes beyond soil, such as the use of personal care products or age-related behavioural changes, may play a more important role in this age group.

### 3.6. “Reference Values” of Ag in Hair

Potential reference values for Ag in hair were calculated following IUPAC recommendations [22], using the 95th percentile of the population distribution with a 95% confidence interval (CI-PP95; Table 1). For children, the CI-PP95 was 0.2866–0.5383 µg/g, with sex-specific intervals of 0.2635–1.0817 µg/g for females and 0.1758–0.3582 µg/g for males. Although the central tendency values (median and geometric mean) were slightly lower in adolescents compared with children, the CI-PP95 intervals were notably higher in adolescents (0.5248–3.0795 µg/g vs. 0.2866–0.5383 µg/g), with sex-specific intervals of 0.7720–4.2404 µg/g for female adolescents and 0.1600–0.4526 µg/g for male adolescents.

## 4. Discussion

### 4.1. Hair Ag Levels in Children and Adolescents

The Ag concentrations measured in children from Alcalá de Henares fall within the reference range proposed by Ballesteros et al. [8] for young people in Madrid (0.032–1.430 µg/g). However, our children’s values were slightly lower than those reported by Ballesteros et al. [8]: median 0.1120 (range 0.0139–2.6906) µg/g in Alcalá versus 0.251 µg/g (range 0.132–0.491) µg/g for 6–10-year-olds in Madrid. Among adolescents, our median value of 0.0695 µg/g (range 0.0225–9.9268) was also lower than the 0.119 µg/g (range 0.052–0.339) reported for >15-year-olds by these authors. Ballesteros et al. [8] also reported sex-dependent deposition of Ag in hair, with higher levels in females, which aligns with the findings of our study. While various studies have documented elevated levels of Ag (and other metals) in female scalp hair, suggesting sex-related disparities in the assimilation and toxicokinetics of Ag, further investigation is necessary to elucidate this phenomenon. This is particularly relevant given that other authors have reported contrasting trends. For instance, higher levels were observed in 21–22-year-old male individuals from the Lower Silesia Region of Poland (0.229 vs. 0.095 mg/kg [32]), although these differences did not reach statistical significance. This observation has led some researchers to hypothesise that female individuals may be more susceptible to metal exposure than male counterparts, particularly at higher exposures [33,34].

In Alcalá, the levels of Ag detected in children were also lower than those reported in other international studies. For instance, the geometric mean quantified in our cohort (0.1016 µg/g) was slightly lower than that reported in young individuals aged 11–13 years from Iglesias, southwestern Sardinia, an area characterised by numerous abandoned mines (0.13 µg/g [34]). Our children’s levels were also below those described in 8-year-old children living in a polymetallic mining area of the Bolivian Altiplano (0.33 µg/g [35]). However, the presence of Ag in scalp hair in Alcalá’s children was higher when compared with 11–13-year-old adolescents living on the island of Sant’Antioco (0.095 vs. 0.015 µg/g [34]), suggesting that background exposures in Alcalá, even outside heavily contaminated sites, are not negligible.

When considering adolescents in our study, their central tendency values (median 0.0695 µg/g; geometric mean 0.0839 µg/g) were slightly lower than those of children, and the age-group comparison was not statistically significant (*p*-value = 0.153). Moreover, a higher proportion of censored values was observed in adolescent hair compared with children. This may reflect the age-dependent decline in Ag concentrations reported in the literature [8], in which younger children exhibited higher Ag levels than older teenagers. Nevertheless, adolescents showed greater variability, with some female individuals reaching up to 9.9 µg/g. This dispersion could reflect lifestyle factors (cosmetics, hair products, increased use of consumer goods containing Ag nanoparticles) superimposed on environmental exposure. Sensitivity checks excluding these extreme values confirmed that the main statistical outcomes—namely the absence of significant age-related differences and the presence of clear sex-related variability—remained unchanged, supporting the robustness of our conclusions.

Comparable studies of Ag in adolescents are limited. Ballesteros et al. [8] examined children and adolescents aged up to 18 years in Madrid and reported an age-dependent decline in Ag concentrations, with younger children (6–10 years) showing higher levels than older teenagers (>15 years). In contrast, our study did not identify significant age-related differences between children (median 0.1120 µg/g) and adolescents (median 0.0695 µg/g; *p*-value = 0.153). The greater variability observed among adolescents in Alcalá, driven by a small number of elevated values in females, suggests that age alone is not a consistent determinant of hair Ag levels across different populations.

Internationally, our adolescent values are consistent with reference ranges reported in French adults (0.02–1.31 µg/g [36]) and with the reference intervals proposed by Chojnacka et al. [37] for Polish university students (0.036–0.801 µg/g), which remain one of the most robust datasets for young adult populations in Europe. More recent work by Izydorczyk et al. [32] highlighted a striking temporal decline in Ag exposure in Poland, with median hair concentrations decreasing from 0.290 µg/g in 2009 to 0.010 µg/g in 2019 (a −96.6% reduction), attributed to improving environmental conditions. The median Ag in Alcalá adolescents (0.0695 µg/g) therefore lies within the historical reference ranges proposed by Chojnacka et al. [37] and is above the very low levels now observed in contemporary Polish students, suggesting that while Ag exposure in Alcalá remains typical for general European populations, it may be somewhat higher than the most recent benchmarks reported for Poland.

In addition, Momčilović et al. [6] reviewed environmental human silver exposure and highlighted that hair is a useful biomarker of cumulative Ag exposure, though it is strongly influenced by external sources such as personal care products, jewellery, and household items. This is consistent with our findings of high outlier values in female adolescents, which are unlikely to be explained by soil exposure and may instead reflect lifestyle-related pathways. Together, these international comparisons and reviews confirm that Ag levels in Alcalá children and adolescents are representative of background exposure in general populations without occupational sources, while underlining the importance of considering consumer-product and environmental factors that may disproportionately affect subgroups, particularly females. Overall, these results align with global biomonitoring evidence, indicating that although Ag exposure is widespread, concentrations in Spain remain comparatively low.

At the international level, evidence on silver concentrations in human hair remains scarce but indicates substantial geographical variability. Studies from Asia, Eastern Europe, and North America have reported higher Ag levels than those observed in Spain, frequently linked to industrial or urban pollution, cosmetic use, or occupational exposure [6,32,34,35]. In contrast, Spanish cohorts [including those from Madrid [8] and Alcalá de Henares (present study)] show generally low background concentrations, consistent with non-occupational exposure in urban environments. These findings underscore the importance of establishing local biomonitoring data to characterise baseline exposures and support the early detection of emerging Ag-related risks in Europe.

It should be noted that comparisons with previously published studies are intended solely to contextualise our findings within the limited international dataset available for Ag in human hair. Given methodological differences in sampling, washing, and analytical protocols, these comparisons should not be interpreted as direct equivalences but rather as indicative of background exposure ranges.

### 4.2. Sex, Residential Area and Environmental Contributions

In Alcalá, Ag levels were consistently higher in females compared with males, both in children (*p*-value = 0.015) and adolescents (*p*-value = 0.002). This sex-related pattern has also been reported in Madrid [8] and in other trace element studies, suggesting differences in hair deposition linked to biological and behavioural factors. While some authors have hypothesised that women may be more susceptible to metal exposure than men, especially in higher-exposure contexts [33,34], other studies have shown contrasting trends. For instance, Izydorczyk et al. [32] found no statistically significant sex differences in Ag among Polish students, although hair dyeing increased Ag by more than 10-fold, underscoring the potential role of cosmetic practices in modulating hair levels. Similarly, Galizia et al. [5] showed that dyed hair can accumulate higher concentrations of potentially toxic elements, while Chojnacka et al. [38] demonstrated that wearing silver jewellery was associated with approximately threefold higher hair Ag compared with non-wearers. In our study, only the proximal 1 cm of untreated scalp hair was analysed, and samples were collected exclusively from individuals who reported never having used hair dyes or other chemical treatments. This, together with a rigorous cleaning protocol, would minimise any effect of hair length on the observed sex-related differences. These observations could therefore suggest that external contamination and lifestyle practices may contribute to the higher and more variable Ag values detected among female adolescents in Alcalá. In addition, sex-specific physiological changes during adolescence, including hormonal fluctuations that might influence metal incorporation into hair, could also play a role, as suggested by trace-element differences reported between male and female adolescents in previous Spanish cohorts [8]. Given the weak correlations between hair and soil Ag, these findings point towards alternative exposure routes that are more consistent with lifestyle- and consumer-related factors.

Accordingly, beyond soil exposure, several lifestyle- and consumer-related factors could plausibly account for the higher and more variable Ag levels observed in female adolescents. Although only untreated, natural hair was analysed, regular use of personal care and hygiene products (e.g., shampoos, creams, deodorants, and sprays) that incorporate Ag or Ag-nanoparticles for their antimicrobial properties may lead to surface deposition or dermal absorption [3,6,39]. Direct contact with silver jewellery has been shown to increase Ag content in hair by roughly threefold compared with non-wearers [38], while the expanding use of Ag-based coatings and textiles in clothing, sportswear, and medical or household materials offers additional potential exposure routes through skin contact or indoor dust resuspension [2,3]. Low-level dietary intake may also contribute, for example, through food contact materials or supplements containing colloidal silver, although its relative importance remains uncertain [39,40].

Collectively, these lifestyle pathways provide plausible explanations for the observed sex differences and outlier values and align with recent reviews highlighting that consumer-product exposure is now a dominant driver of silver uptake in non-occupational populations [6,39]. Future biomonitoring efforts should therefore integrate questionnaires addressing jewellery use, product consumption, and dietary habits to better apportion exposure sources.

By simultaneously assessing children and adolescents within the same urban setting, this study could provide new insight into age- and sex-related variability in Ag exposure under shared environmental conditions. The concurrent inclusion of topsoil data may also help to contextualise human exposure within local environmental Ag distribution, an approach that has rarely been applied in previous biomonitoring studies. Together, these aspects may enhance understanding of baseline Ag exposure patterns in young populations and contribute to the development of future human biomonitoring strategies.

Regarding residential areas, no significant differences were observed among children (*p* = 0.172). In adolescents, the updated analysis confirmed significant geographical variation (*p* = 0.0004), with Zone I (low-population-density, greener neighbourhoods) showing significantly lower Ag levels than Zones II (high-population-density) and III (traffic-dense areas), whereas no significant differences were found with the industrial Zone IV. This pattern suggests that Ag exposure may be slightly higher in compact urban areas and districts influenced by road traffic, while remaining comparatively lower in suburban and greener environments. Similar trends have been reported for other trace elements; He et al. [41] observed significantly higher Cd, Cu, Ni, and Pb concentrations in the hair of urban versus rural residents in Chongqing (China), reinforcing that urban living may increase metal exposure through multiple pathways. Although silver was not included in that study and extrapolation must be made with caution, these findings support the influence of urban factors. Additionally, as many participants may attend schools outside their residential neighbourhoods, exposure near schools (potentially different from that at home) could introduce variability and uncertainty in our results. Future studies should therefore monitor both residential and school environments to better characterise the spatial determinants of Ag exposure in youth populations.

As shown in the results section, correlations between Ag in hair and Ag in topsoils were weak and non-significant, indicating that soils are not a primary determinant of Ag exposure in Alcalá. Instead, alternative pathways, including inhalation of airborne particles, indoor dust, dietary intake, and consumer product use, likely account for the observed inter-individual variability. Localised patterns in Zone II, where higher Ag in hair was observed, may point to a partial contribution of soils in certain micro-environments, but the overall evidence suggests that lifestyle- and product-related sources are more influential than environmental background contamination.

Additionally, we should acknowledge that individuals in our study might attend schools located in neighbourhoods with different characteristics from those of their residential areas. It is therefore possible that Ag exposure around their homes differed significantly from that in their school environments, which may partially explain the low correlations observed between hair and soil samples in our study. Future research should assess silver in both school and household environments to better characterise daily exposure across young people’s activity spaces. From a health risk perspective, hazard quotients derived from topsoil exposure were <1 for both children and adults [24], supporting the conclusion of minimal toxic risk from this pathway. Nonetheless, the higher dispersion observed in adolescents underlines the need for further research on exposure pathways. Because there are limited data available to support the derivation of a quantitative screening value for silver in human hair [1], the concentrations reported here cannot be directly interpreted in terms of health risk and should instead be considered as indicators of environmental and lifestyle exposure. Nevertheless, evidence from other populations highlights that extreme outliers may be clinically relevant. In a study of 311 adults from Zagreb, hair Ag concentrations ranged from 0.00006 to 7.35 µg/g (median 0.0695 µg/g), with women showing higher median values than men (0.077 vs. 0.050 µg/g) [6]. Notably, three individuals with hair Ag levels above 4 µg/g exhibited neurological and psychiatric symptoms, including peripheral neuropathy and depressive disorders, suggesting that very high Ag burdens can have adverse health effects [6]. In this context, our observation of several female adolescents in Alcalá with elevated Ag values, which widened the upper bound of their CI-PP95 (Table 1), underlines the importance of identifying and monitoring such subgroups. Continuous biomonitoring of silver in children and adolescents is therefore essential, both to detect unusual exposures linked to lifestyle or consumer products and to provide robust local benchmarks that can inform future public health interventions.

### 4.3. Reference Values

The reference intervals calculated according to IUPAC recommendations showed that, although adolescents in Alcalá had slightly lower central tendency values than children, their CI-PP95 was markedly higher. This apparent discrepancy reflects the greater variability and the presence of several high values, particularly among female adolescents, which substantially widened the upper bound of the reference interval. At the same time, nearly one-fifth (19.6%) of adolescent samples were below the limit of detection, in contrast to none of the children’s samples. Taken together, these findings suggest that while many adolescents exhibited very low or undetectable Ag levels, a smaller subgroup showed markedly elevated concentrations. This pattern may reflect heterogeneous lifestyle-related exposures during adolescence, as discussed previously. Although adolescents in Alcalá exhibited lower central tendency values than children, the difference was not statistically significant, highlighting that local exposure patterns may not follow the age-related decline described in other populations.

These values constitute the first reference intervals for Ag in children and adolescents from Alcalá de Henares, complementing the ranges previously proposed for Madrid [8], and providing a local benchmark for future biomonitoring. Accordingly, the reference values reported here should be interpreted solely as population exposure benchmarks, and not as health-based guidance values, since no toxicological thresholds for silver in hair have yet been established [1].

### 4.4. Study Limitations and Strengths

This study has some limitations. It was conducted in a single urban area (Alcalá de Henares), which may restrict generalisability to other regions. Information on dietary habits, use of cosmetics or jewellery, and other potential sources of silver exposure was not collected, limiting the ability to identify specific contributors to variability. Future studies with larger and more balanced samples should apply multivariate regression models to disentangle the independent effects of age, sex, and environmental or behavioural variables on Ag levels. The present analysis focused on descriptive statistics and reference interval derivation, as covariate data were not available to support multivariate modelling. In addition, hair is susceptible to external contamination despite rigorous washing protocols, and there are currently no health-based screening values for silver in hair [1], so the reference intervals reported here should be interpreted as population benchmarks rather than direct indicators of health risk. Future studies should include more recent and multi-centre sampling, incorporate additional biological matrices, and collect detailed information on potential exposure determinants to strengthen interpretation.

Despite these limitations, this study also has several strengths. It includes participants from neighbourhoods representing distinct land-use types (residential, high-density urban, traffic-influenced, and industrial) allowing for a spatially differentiated assessment of exposure patterns within the same city. Moreover, robust statistical methods specifically designed to handle censored environmental data were applied, ensuring reliable interpretation of concentrations below detection limits. This analytical approach provides a methodological framework that may inform and strengthen future human biomonitoring studies dealing with similar data characteristics.

## 5. Conclusions

The present study provides the first reference intervals for silver in hair from healthy, non-occupationally exposed children and adolescents residing in Alcalá de Henares, a major urban area in Madrid region, Spain. Ag concentrations were generally low and within the range reported for background populations, indicating that current exposures in Alcalá de Henares are unlikely to pose a risk to health. Although central tendency values were slightly lower in adolescents than in children, the wider CI-PP95 observed in adolescents reflects greater inter-individual variability, driven by several high values in females. This heterogeneity, together with a higher proportion of samples below the detection limit in adolescents, suggests that exposures during adolescence are more variable and likely influenced by lifestyle factors such as cosmetics, jewellery, or consumer products containing Ag nanoparticles, rather than by soil contamination.

Sex-related differences were consistent across both age groups, with females showing significantly higher Ag levels than males, a pattern also described in other populations but still poorly understood from a mechanistic perspective. These differences may have clinical significance and require further investigation. Importantly, the establishment of local reference values for Ag in hair provides a valuable baseline for future human biomonitoring programmes in Spain and Europe. These data will support temporal trend analyses, help identify potential emerging sources of exposure linked to Ag nanoparticles, and inform public health surveillance and risk assessment frameworks. Taken together, our findings suggest that Ag exposure in Alcalá reflects background levels typical of non-occupationally exposed European populations, but with meaningful variability linked to age, sex, and lifestyle. Continuous biomonitoring of silver in sensitive groups such as children and adolescents is recommended to track changes in exposure linked to the increasing use of Ag nanoparticles in consumer products and guide evidence-based preventive strategies.

## Figures and Tables

**Table 1:** Levels of Ag in hair (µg/g) of children and adolescents from Alcalá de Henares, Spain.

Population		LoD	% < LoD	Arithmetic Mean	Geometric Mean	Median	Range	Interquartile Range	P95	CI-PP95
Children	Female	0.0036	0	0.1912 ± 0.3387 *	0.1201	0.1205	0.0168–2.6906	0.0747, 0.1867	0.4577	0.2635–1.0817
	Male		0	0.1082 ± 0.0804	0.0848	0.0892	0.0139–0.3841	0.0436, 0.1402	0.3058	0.1758–0.3582
	Total		0	0.1566 ± 0.2668	0.1016	0.1120	0.0139–2.6906	0.0623, 0.1679	0.3504	0.2866–0.5383
Adolescents	Female	0.0224	16.2	0.5401 ± 1.4498 **	0.1200	0.1057	0.0225–9.9268	0.0346, 0.4215	2.3554	0.6547–6.0749
	Male		27.6	0.0762 ± 0.1021	0.0401	0.0387	0.0273–0.5023	0.0224, 0.0606	0.3071	0.0606–0.3071
	Total		19.6	0.4010 ± 1.2310	0.0839	0.0695	0.0225–9.9268	0.0308, 0.2487	1.3401	0.5248–3.0795

LoD = limit of detection (µg/g); % < LoD = percentage below limit of detection. Arithmetic mean results are presented as mean values ± S.D. Statistical significance: * (*p*-value = 0.015), ** (*p*-value = 0.002); P95 = 95th percentile; CI-PP95 = 95% confidence interval for 95PP (population percentile).

**Table 2:** Levels of Ag in hair (µg/g) of children and adolescents from Alcalá de Henares, according to the zones.

Population	Zone	*n*	% < LoD	Mean	SD	LCL Mean	UCL Mean	Median	*p*-Value
Children	I	24	0	0.1076	0.1042	0.0636	0.1516	0.0778	0.172
	II	60	0	0.1963	0.3648	0.1020	0.2905	0.1201	
	IV	36	0	0.1232	0.0714	0.0991	0.1474	0.1077	
Adolescents	I	36	36.11	0.1333 **^a^**	0.1927	0.0665	0.1948	0.0320	0.0004
	II	20	5.0	1.2930 **^b^**	2.4490	0.1157	2.4682	0.2145	
	III	24	4.17	0.2095 **^b^**	0.2865	0.0859	0.3324	0.0887	
	IV	17	23.53	0.1916 **^ab^**	0.3136	0.0237	0.3545	0.0695	

*n* = number of hair samples collected. Different superscript letters within the same column indicate statistically significant differences between zones. % < LoD = percentage of samples below the limit of detection. LCL Mean = lower 95% confidence limit of the mean; UCL Mean = upper 95% confidence limit of the mean.

## Data Availability

Data for this article, including data on hair and topsoils, are available at the Open Science Framework (OSF) at https://osf.io/hmyae/?view_only=6c8a9d4b34f54efda007d71789c078c8 (accessed on 16 November 2025).
